# Feasibility and Safety of Local Treatment with Recombinant Human Tissue Factor Pathway Inhibitor in a Rat Model of *Streptococcus pneumoniae* Pneumonia

**DOI:** 10.1371/journal.pone.0127261

**Published:** 2015-05-18

**Authors:** Florry E. van den Boogaard, Jorrit J. Hofstra, Cornelis van ‘t Veer, Marcel M. Levi, Joris J. T. H. Roelofs, Tom van der Poll, Marcus J. Schultz

**Affiliations:** 1 Laboratory of Experimental Intensive Care and Anesthesiology (L·E·I·C·A), Academic Medical Center, University of Amsterdam, Amsterdam, The Netherlands; 2 Department of Intensive Care Medicine, Academic Medical Center, University of Amsterdam, Amsterdam, The Netherlands; 3 Center for Experimental and Molecular Medicine (CEMM), Academic Medical Center, University of Amsterdam, Amsterdam, The Netherlands; 4 Department of Internal Medicine, Academic Medical Center, University of Amsterdam, Amsterdam, The Netherlands; 5 Center for Infection and Immunity Amsterdam (CINIMA), Academic Medical Center, University of Amsterdam, Amsterdam, The Netherlands; 6 Department of Pathology, Academic Medical Center, University of Amsterdam, Amsterdam, The Netherlands; 7 Department of Infectious Diseases, Academic Medical Center, University of Amsterdam, Amsterdam, The Netherlands; University of Kentucky, UNITED STATES

## Abstract

Pulmonary coagulopathy is intrinsic to pulmonary injury including pneumonia. Anticoagulant strategies could benefit patients with pneumonia, but systemic administration of anticoagulant agents may lead to suboptimal local levels and may cause systemic hemorrhage. We hypothesized nebulization to provide a safer and more effective route for local administration of anticoagulants. Therefore, we aimed to examine feasibility and safety of nebulization of recombinant human tissue factor pathway inhibitor (rh-TFPI) in a well-established rat model of *Streptococcus (S*.*) pneumoniae* pneumonia. Thirty minutes before and every 6 hours after intratracheal instillation of *S*. *pneumonia* causing pneumonia, rats were subjected to local treatment with rh-TFPI or placebo, and sacrificed after 42 hours. Pneumonia was associated with local as well as systemic activation of coagulation. Nebulization of rh-TFPI resulted in high levels of rh-TFPI in bronchoalveolar lavage fluid, which was accompanied by an attenuation of pulmonary coagulation. Systemic rh-TFPI levels remained undetectable, and systemic TFPI activity and systemic coagulation were not affected. Histopathology revealed no bleeding in the lungs. We conclude that nebulization of rh-TFPI seems feasible and safe; local anticoagulant treatment with rh-TFPI attenuates pulmonary coagulation, while not affecting systemic coagulation in a rat model of *S*. *pneumoniae* pneumonia.

## Introduction

Pneumonia is associated with a local procoagulant state due to enhanced activation of coagulation, reduced anticoagulant capacity and inhibition of fibrinolysis in the alveolar compartment [1–3]. Intrapulmonary fibrin deposition and inflammation-induced coagulopathy aggravate lung injury and ultimately compromise pulmonary function [4]. As activation of coagulation is both a consequence and a contributor to ongoing lung injury, pulmonary coagulopathy has been suggested as a target for therapeutic intervention in patients with pneumonia [1].

Tissue factor (TF) is appreciated as the main initiator of coagulation with infection. Results from animal and human studies suggest that pathological expression of TF by inflammatory cells plays a detrimental role on the outcome of community-acquired pneumonia (CAP) [3, 5, 6]. At the same time, tissue factor pathway inhibitor (TFPI), the endogenous inhibitor of the TF pathway, is overwhelmed by increased TF procoagulant activity [7, 8]. In several models of lung injury it is shown that blocking the TF pathway during lung injury prevents local coagulation and preserves lung function [9–11]. Moreover, systemic TF inhibition reduces pulmonary and systemic coagulation in rodent models of pneumonia [5, 12, 13].

One important drawback of systemic administration of anticoagulants is the increased risk of bleeding complications [14–16]. In addition, it is uncertain how well the systemically administered drugs penetrate into lung tissue during pneumonia [17]. Notably, TFPI in pulmonary edema fluid in patients with the acute respiratory distress syndrome (ARDS) has been shown to be truncated and inactive [7]. Considering these issues, local treatment with anticoagulant agents is an appealing strategy as local treatment may be safer, while at the same time potentially leading to higher alveolar concentrations [18].

There are no preclinical studies that have investigated the effects of local treatment with TFPI in pneumonia so far. We here hypothesize that local administration of recombinant human (rh)-TFPI by means of nebulization efficiently attenuates pulmonary coagulopathy while leaving systemic coagulation unaffected. Therefore we infected healthy rats with *Streptococcus pneumoniae*, the most common causative pathogen of CAP [19], and treated them locally with rh-TFPI or placebo.

## Material and Methods

### Animals

The Institutional Animal Care and Use Committee of the Academic Medical Center of the University of Amsterdam approved all experiments. Animals were handled in accordance with the guidelines prescribed by the Dutch legislation, and international guidelines on protection, care, and handling of laboratory animals. Male Sprague—Dawley rats (250–300 g; 8–10 weeks old) (Harlan, The Hague, The Netherlands) were allowed to acclimatize to laboratory conditions for at least 7 days (12:12 h day—night cycle at 22°C) before handling.

### Study design

Pneumonia was induced by intratracheal instillation under light sedation with 5% isoflurane of 8,5 x 105 colony-forming units (CFU) *S*. *pneumoniae* (serotype 3, ATCC 6303) in a total volume of 250 μL of bacterial suspension, which was cultured as described previously [12, 13]. Rats were randomized to nebulization with rh-TFPI 10 mg/kg (Tifacogin, Novartis, Chiron, Emeryville, CA) or vehicle (300 mM L-arginine, 20 mM trisodium citrate dihydrate, pH 5.5) (n = 7 per group). Uninfected rats were nebulized with vehicle (n = 3) to evaluate the effect of nebulization alone. Uninfected untreated rats served as naïve controls (n = 5). Per group rh-TFPI or vehicle was administered by nebulization in a total volume of 5 mL at 30 minutes before and every 6 hours after induction of pneumonia. The dosing strategy was based on data from previous studies with rh-TFPI administered intravenously in *S*. *pneumoniae* pneumonia [12] and an estimation of the efficacy provided by the nose-only exposure system, as described below.

### Nebulization

For local treatment with rh-TFPI we used an adapted dynamic airflow, nose-only exposure system, which allows direct exposure of nebulized agents to the noses of the animals, as described before [20]. In short, this system consists of a concentric manifold connected to the necks of bottle-like restraint tubes (CHT 249 restraint tube, CH technologies Inc., Westwood, New Jersey) in which the animals were confined with their noses adjacent to the bottlenecks. The bottles are detachable allowing disassembly of the device for cleaning. The inhalation chamber is suitable to accommodate up to 7 rats at once. The aerosolized agent was supplied to the upper end of the manifold, flowed adjacent to the noses of the individual animals, and then was drawn out through the bottom of the manifold. The aerosol atmosphere was generated using the AeronebPro Micropump Nebulizer (Aerogen Ltd.). The Aeroneb Pro Nebulizer uses a vibrating mesh with multiple apertures to generate a fine—particle, low—velocity aerosol and produces aerosols with an average size of 2.1 μm. At a constant oxygen flow (2 L/min) the aerosols were directed to the inhalation chamber. The animals were accommodated to restraint tubes at several occasions in the week before the experiments.

### Blood and tissue sampling

At 42 hours after induction of pneumonia, rats were sacrificed with an intramuscular injection of ketamine 45 mg/kg (Eurovet, Bladel, The Netherlands) and medetomidine 0.25 mg/kg (Novartis, Arnhem, The Netherlands). Blood was collected from the inferior vena cava in citrated (0.109 M) vacutainer tubes. The right lung was ligated, and the left lung was lavaged three times with 2 mL ice—cold saline, 0.3% BSA, 1 mM EDTA. The right superior lobe was fixed in 10% buffered formalin and embedded in paraffin. The remaining lung lobes were weighed and homogenized in 4 volumes (i.e., 4 x lung weight in μL) of sterile saline using a tissue homogenizer (Biospec Products, Bartlesville, OK).

### Measurements

Plasma and cell—free supernatants from bronchial lavage fluids (BALF) were used for measuring levels of rh-TFPI, TFPI activity and coagulation. Total cell numbers in lavage fluid were determined using an automated cell counter (Z2 Coulter Pariticle Counter, Beckman Coulter Corporation, Hialeah, FL). Neutrophil counts in lavage fluids were performed on cytospin preparations stained with a modified Giemsa stain (Diff-Quick; Dade Behring AG, Düdingen, Switzerland). Commercially available ELISA’s were used to measure levels of tumor necrosis factor (TNF)-α, interleukin (IL)-6, and cytokine induced neutrophil chemoattractant (CINC)-3 (all R&D Systems, Abingdon, United Kingdom) and myeloperoxidase (MPO; HyCult biotechnology b.v., Uden, The Netherlands).

To quantify bacterial numbers in lungs and blood, serial ten—fold dilutions of lung homogenates, lavage fluid and whole blood were made in sterile isotonic saline and plated onto sheep—blood agar plates. After 16 hours of incubation at 37°C in 5% CO_2_, the numbers of CFU were counted.

#### Assays

To determine the efficacy of local delivery of rh-TFPI, by measuring total rh-TFPI immunogen levels in the lung and in plasma of rats nebulized with rh-TFPI, we developed an enzyme-linked immunosorbent assay (ELISA) using monoclonal mouse anti-human TFPI directed against the Kunitz domain 2 (Sanquin, Amsterdam, the Netherlands) as a coating antibody and polyclonal rabbit anti-human TFPI (kind gift of Dr. Walter Kisiel, University of New Mexico, Albuquerque NM, USA) as a detecting antibody.

Furthermore, to determine if nebulization with rh-TFPI affected the overall systemic TFPI activity, we employed the two-stage chromogenic TFPI assay originally described by Sandset et al by measuring its inhibitory activity in a factor Xa-generation assay [21]. In brief, heat—inactivated plasma was incubated with a mixture of recombinant FVIIa (Novoseven, Novo Nordisk A/S, Bagsvaerd, Denmark), a limited amount of FX (kind gift of Dr. Walter Kisiel, University of New Mexico, Albuquerque NM, USA) and relipidated recombinant TF (Innovin, Dade Behring, Surrey, UK) in order to form TF-FVIIa-FXa-TFPI complexes. To measure residual TF activity excess FX was added in the second stage and FXa generation was determined using S2222. Standard curves were prepared by serial dilution of citrated normal rat plasma.

Thrombin—antithrombin complexes (TATc) and fibrin degradation products (FDP) were measured using commercially available ELISA (TATc: Behringwerke AG, Marburg, Germany, FDP; Asserachrom D—Di, Diagnostica Stago, Asnières—sur—Seine, France); antithrombin (AT), plasminogen activator activity (PAA), and plasminogen activator inhibitor (PAI)-1 activity were measured by automated amidolytic assays [22].

### Histopathology

Immediately after rats were killed, lung samples were fixed in 10% buffered formalin for 24h and embedded in paraffin in a routine fashion. Four—micrometer sections were stained with hematoxylin and eosin (H&E). All slides were coded and scored for the following parameters: interstitial inflammation, endothelialitis, bronchitis, edema, pleuritis and thrombus formation, and bleeding by a pathologist who was blinded for group identity. Confluent (diffuse) inflammatory infiltrate was quantified separately and expressed as percentage of the lung surface; the number of thrombi was counted in 5 random microscopic fields. The remaining parameters were rated separately on a scale from 0 (condition absent) to 4 (present in massive amounts).

### Statistical analyses

Comparisons between the experimental rat groups and vehicle—treated placebo rat group were performed using Kruskal—Wallis tests, followed by Mann—Whitney *U*—tests where appropriate. Data are expressed as individual data or as median with interquartile ranges. A *p*-value < 0.05 was considered statistically significant. Statistical analyses were performed with GraphPad Prism (GraphPad Software, San Diego, CA).

## Results

### Levels of rh-TFPI in BALF and plasma

**Fig 1:**
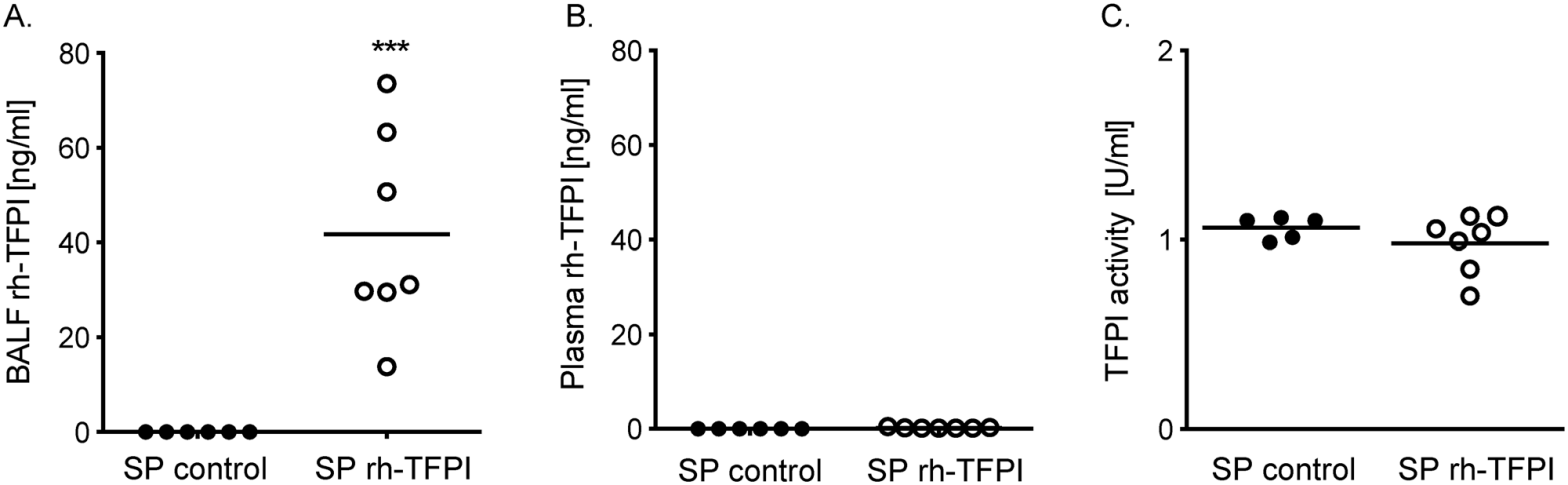
Nebulization with rh—TFPI increases levels of rh-TFPI in bronchoalveolar lavage fluid but not in plasma. Levels of rh-TFPI measured in bronchoalveolar lavage fluid (BALF) (A), and plasma (B), and TFPI activity in plasma (C) after nebulization of rh-TFPI (rh-TFPI) or vehicle (control) in rats 42 hours after intratracheal instillation of *S*. *pneumoniae*. Data are represented as individual data with median. ***p < 0.001 versus control.

In previous studies nebulization of anticoagulants attenuated pulmonary coagulopathy, but also affected systemic coagulation [18]. To verify whether rh-TFPI delivery by nebulization was restricted to the lung compartment, we measured rh-TFPI levels in lavage fluid and plasma. Rh-TFPI levels were significantly increased in lavage fluid of rh-TFPI treated rats, and no rh-TFPI was detected in lavage fluid of vehicle treated rats (Fig 1A). Rh-TFPI levels remained undetectable in plasma of rh-TFPI treated rats (Fig 1B), confirming containment of rh-TFPI delivery within the lung compartment when nebulized. Systemic TFPI activity was also not altered by nebulization of rh-TFPI (Fig 1C), further suggesting local administration of rh-TFPI does not have a systemic effect.

### Pulmonary and systemic activation of coagulation

**Fig 2:**
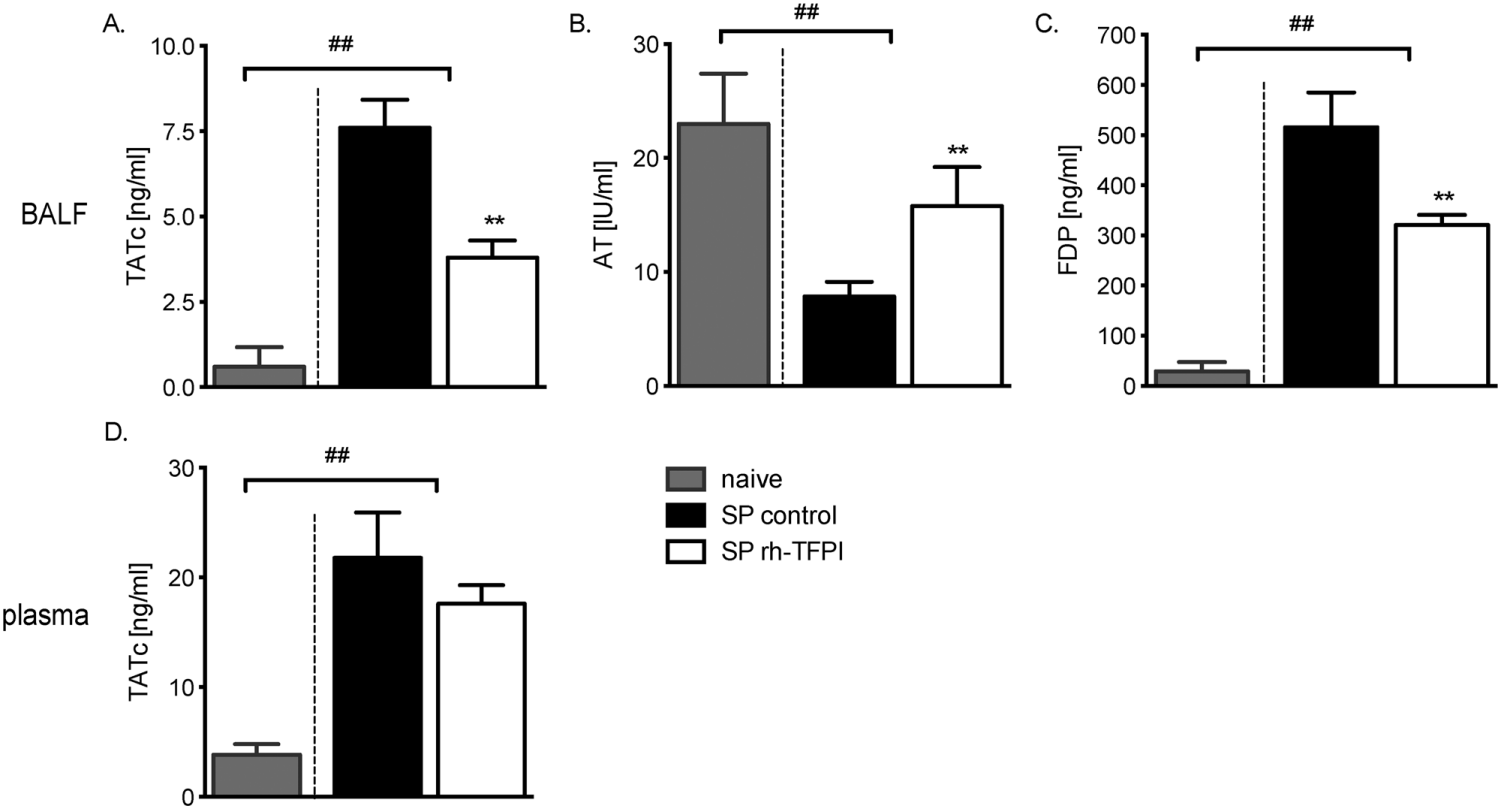
Nebulization with rh-TFPI attenuates coagulation in BALF but not in plasma. Levels of thrombin-antithrombin complexes (TATc) (A), antithrombin activity (AT) (B), fibrin degradation products (FDP) (C) measured in BALF, and levels of TATc measured in plasma (D) of rats nebulized with rh-TFPI (SP rh-TFPI, open bars) or vehicle (SP control, black bars) 42 hours after intratracheal instillation of *S*. *pneumoniae* and in naïve control rats (control, grey bars). Bars depict median ± IQR; **p < 0.01 versus SP control, ##p < 0.01 versus naïve controls.

Pneumonia is associated with an increase in coagulation activity [2, 3]. Accordingly, we demonstrated a procoagulant state in infected rats as reflected by increased levels of TATc and FDP with a concurrent drop in AT levels in BALF (Fig 2A–2C) and elevated TATc levels in plasma (Fig 2D). Systemic treatment with rh-TFPI is known to reduce activation of coagulation in both pre-clinical and clinical pneumonia studies [9, 12, 13, 23]. Nebulization of rh-TFPI significantly attenuated the infection-induced increase in TATc and FDP levels and reduction of AT levels in lavage fluid compared to rats treated with vehicle (Fig 2A–2C). At the same time, nebulization of rh-TFPI did not alter systemic coagulation (Fig 2D). No differences in coagulation were observed between uninfected naïve and uninfected vehicle nebulized rats (data not shown). Together, these data confirm the efficacy of nebulized rh-TFPI to attenuate pulmonary coagulopathy in pneumonia and suggest that local administration of rh-TFPI does not pose a risk of systemic hemorrhage.

### Pulmonary and systemic fibrinolysis

**Fig 3:**
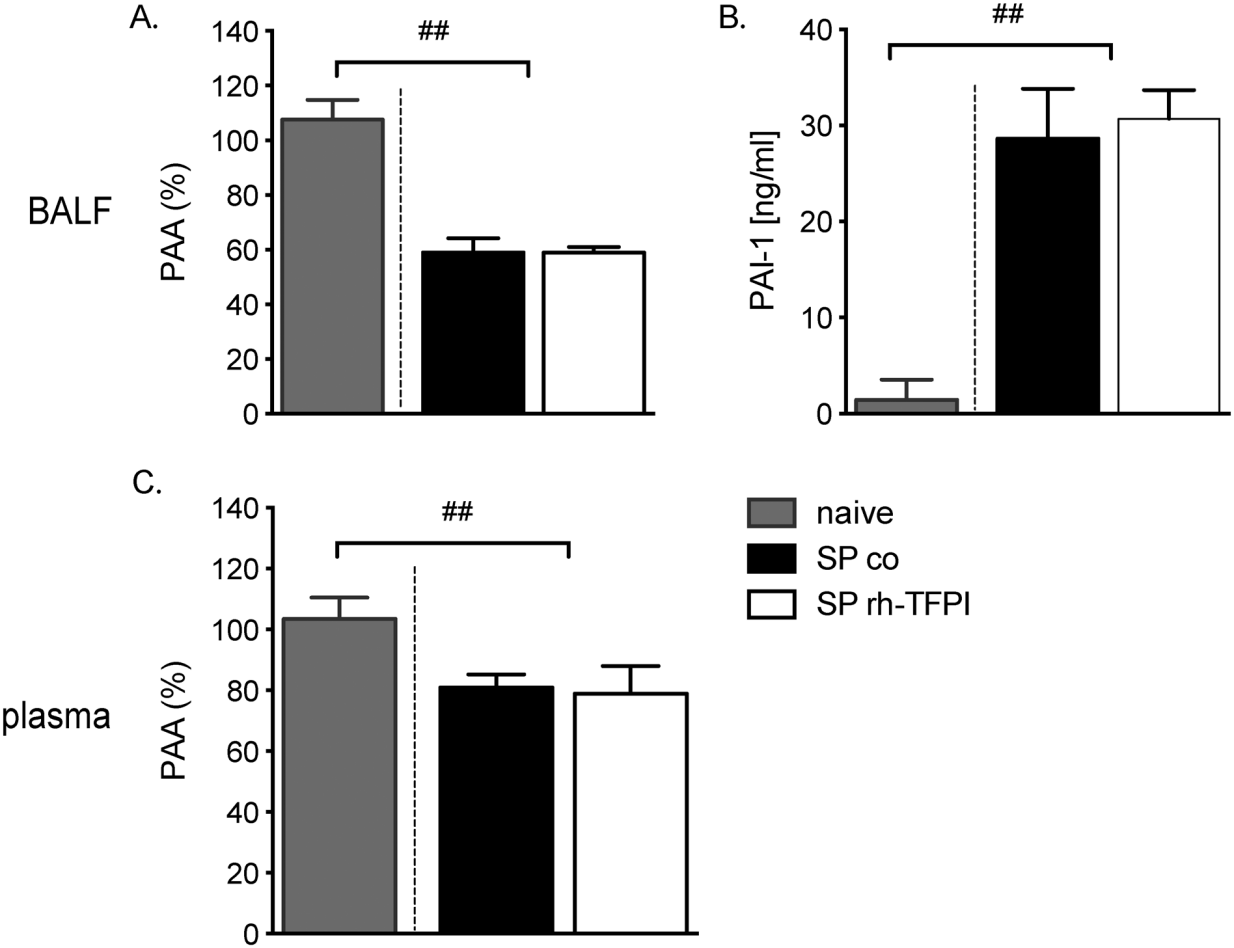
Nebulization with rh-TFPI does not influence local or systemic fibrinolysis. Plasminogen activator activity (PAA) and plasminogen activator inhibitor (PAI)-1 activity in BALF (A and B) and PAA in plasma (C) in rats nebulized with rh-TFPI (SP rh-TFPI, open bars) or vehicle (SP control, black bars) 42 hours after intratracheal instillation of *S*. *pneumoniae* and in naïve control rats (control, grey bars). Bars depict median ± IQR; ##p < 0.01 versus naïve controls.

The used model of pneumococcal pneumonia is associated with inhibition of fibrinolysis due to enhanced release of PAI-1 [12, 20], which resembles changes in patients with pneumonia [2, 24, 25]. In accordance, we measured elevated levels of PAI-1 in BALF and decreased levels of PAA in BALF and in plasma. Nebulization with rh-TFPI did not influence local or systemic fibrinolysis (Fig 3). No differences in fibrinolysis were observed between uninfected naïve and uninfected vehicle treated rats (data not shown).

### Pulmonary and systemic bacterial loads

**Fig 4:**
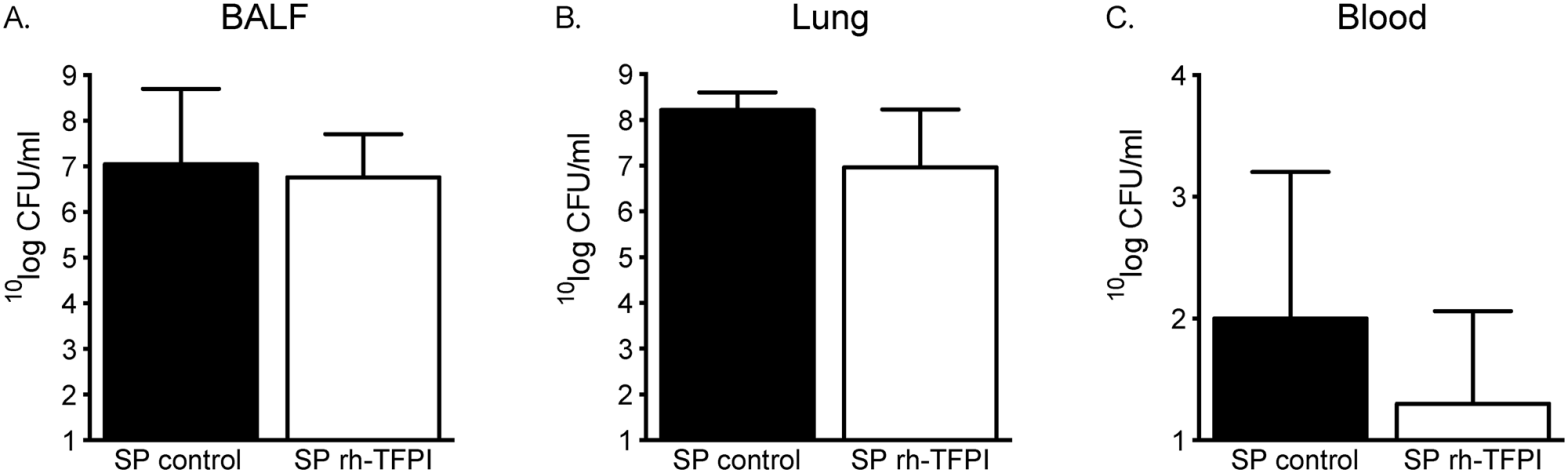
Nebulization with rh-TFPI does not influence bacterial loads in the pulmonary or systemic compartment. Numbers of *S*. *pneumoniae* colony forming units (CFU) were quantified in bronchoalveolar lavage fluid (BALF) (A), lung homogenates (B), and whole blood (C) of rats nebulized with rh-TFPI (rh-TFPI) or vehicle (control) 42 hours after intratracheal instillation of *S*. *pneumoniae*. Bars depict median ± IQR. No statistical difference between groups was observed.

To examine the effect of nebulized rh-TFPI on local bacterial loads and dissemination we quantified bacterial numbers in BALF, lung homogenate and whole blood. Bacterial loads were not different between the study groups (Fig 4).

### Inflammatory response

**Fig 5:**
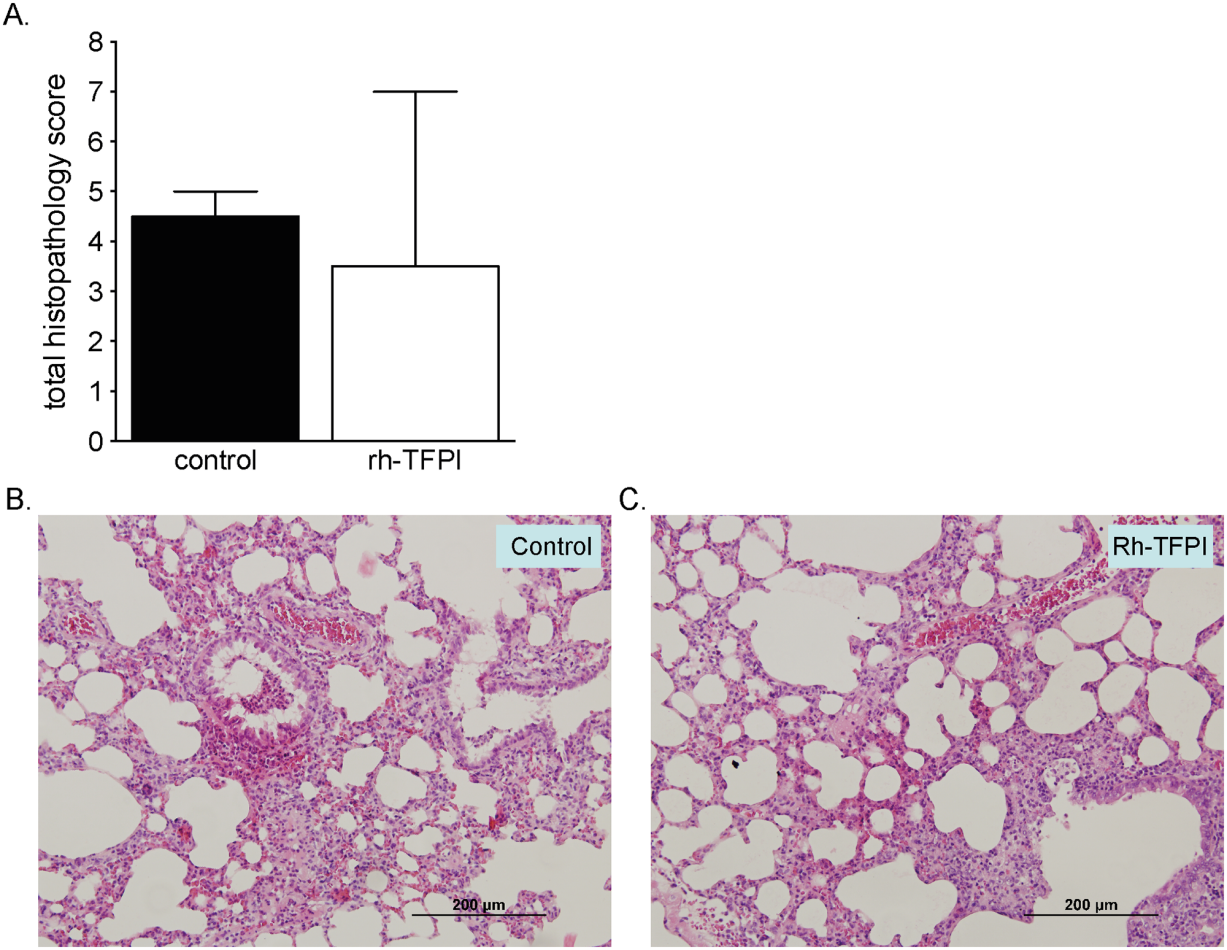
Effects of nebulized rh-TFPI on histopathology. Total histopathology (PA) scores of lung tissue (A) from rats nebulized with rh-TFPI (rh-TFPI) or vehicle (control) 42 hours after intratracheal instillation of *S*. *pneumoniae* with representative microphotographs (B and C). No bleedings were observed. Bars depict median ± IQR. Scale bar = 200 μm. No statistical difference was observed between groups.

A pulmonary inflammatory response was elicited by pneumococcal pneumonia 42 hours after intratracheal challenge with *S*. *pneumoniae* in the lungs of rats, as shown by a significant increase in cell influx in BALF, mainly consisting of neutrophils compared to uninfected control rats (Table 1) and the presence of interstitial inflammation, bronchitis and edema seen in lung histopathology slides represented as total histopathology scores (Fig 5). Inhalation of nebulized rh-TFPI did not affect total cell numbers or absolute or relative neutrophil counts in BALF. However the level of MPO, a marker of neutrophil activity, was reduced in BALF of rats nebulized with rh-TFPI compared to rats treated with vehicle (p = 0.03, Table 1). Furthermore, lung weight and cytokines/chemokines (TNF-α, IL-6, CINC-3) were not influenced by nebulization of rh-TFPI (S1 Fig). No differences in cell counts were observed between uninfected naïve and uninfected vehicle treated rats (data not shown). No lung tissue protective effect was seen from nebulization with rh-TFPI compared to vehicle treated rats reflected by unaltered total histopathology scores (Fig 5).

**Table 1:** Influence of nebulized rh-TFPI on lung inflammation and injury during rat *Streptococcus pneumoniae* pneumonia.

	Control	SP control	SP rh-TFPI
Total cells	28 (12–50)	123 (91–451)^##^	112 (72–187)
PMN	0 (0–0)	88 (24–666)^##^	62 (49–143)
MPO [pg/ml]	88 (86–89)	131 (125–150)	87 (73–99)*

NOTE: Total cell and neutrophil (PMN) counts x 104/ml in bronchoalveolar lavage fluid and myeloperoxidase (MPO) levels in lung homogenates of rats nebulized with rh-TFPI (SP rh-TFPI) or vehicle (SP control) 42 hours after intratracheal instillation of *S*. *pneumoniae* (n = 7 per group), or of naïve rats (control, n = 5). Data are expressed as median (interquartile range)

^##^p < 0.01 versus control

*p < 0.05 versus SP control.

### Local Bleeding

To examine the effect of nebulized rh-TFPI on local bleeding, we specifically looked for bleeding in histopathology slides. Nebulization of rh-TFPI did not result in local bleedings, neither in unchallenged rats, nor in rats with pneumonia.

## Discussion

The present study shows that local administration of rh-TFPI by nebulization is feasible and safe in a well-established rat model of *S*. *pneumoniae* pneumonia. Indeed, nebulization of rh-TFPI inhibited bronchoalveolar coagulation, and this treatment did not result in measurable levels of rh-TFPI in plasma, did not affect systemic coagulation, and did not result in local bleedings.

Coagulation is an essential part of host defense that interacts with the inflammation system to mount an adequate response. This was reflected by a local procoagulant state and systemic coagulopathy caused by infection with *S*. *pneumoniae* in the present rat pneumonia model, which is in accordance with observations from previous studies [5, 12, 13, 26]. Notably, these findings resemble the clinical situation, as increased pulmonary coagulation [2, 5, 27] and systemic coagulation abnormalities [28] are common findings in patients with pneumonia.

Coagulation activation is primarily driven by the TF pathway, and, interference with the TF pathway consistently reduces coagulopathy, both in studies of systemic infection and in studies of local infection, like pneumonia [5, 6, 9, 10, 12, 14, 29–36]. Moreover, in previous rodent studies of pneumococcal pneumonia, blocking the TF pathway strongly inhibited alveolar thrombin generation [5, 12, 13]. In these studies, TF pathway inhibitors were administered systemically, bearing the risk of bleeding complications. Recently, studies have been undertaken to explore the feasibility of local delivery of anticoagulant agents and its effectiveness in the pulmonary compartment.

In the present study, we demonstrate that nebulized rh-TFPI attenuated FDP and TATc levels and largely preserved AT levels in lavage fluid, without affecting TATc levels in plasma, indicating that the anticoagulant effect of nebulized rh-TFPI is restricted to the alveolar compartment. Likewise, in other preclinical studies of local treatment with anticoagulant agents, rh-activated protein C, anti-thrombin, and heparin reduced pulmonary coagulation without affecting systemic coagulation, however, nebulized danaparoid also exerted systemic effects on coagulation [26, 37]. Furthermore, local rh-TFPI treatment did not influence fibrinolysis, as reflected by unaltered PAI-1 or PAA levels in lavage fluid and plasma, which is in accordance with earlier reports [13]. Interfering with the procoagulant response elicited by infection has shown inconsistent effects on host defense. In rat models of direct lung injury blocking the TF pathway attenuated vascular leakage, neutrophil influx and levels of cytokines and chemokines [10, 38]. However, a reduced coagulant response may undermine host defense, as low TF mice demonstrated increased lung hemorrhage with concurrent increased inflammation during acute lung injury [39]. In the present study we did not observe lung hemorrhage in either study group and nebulization of rats with rh-TFPI did not importantly affect inflammation or lung pathology, which was in line with previous reports on pneumococcal pneumonia in rodents [5, 12]. Furthermore, thrombi could raise a barrier for bacteria. Although systemic dissemination has been suggested as a potential drawback of anticoagulant treatment in pneumonia [40], local treatment with rh-TFPI did not influence bacterial counts in the lung or systemic compartment.

Large clinical trials have been carried out to study the effect of restoration of impaired anticoagulant pathways as adjunctive treatment in human sepsis [14–16]. Although inflammation-induced coagulation was attenuated by treatment with anticoagulant agents, these studies did not convincingly show a beneficial effect on outcome [41]. The OPTIMIST trial, investigating rh-TFPI in sepsis patients, suggested a protective effect from rh-TFPI in a subgroup of CAP patients, especially when not treated with heparin [14]. TFPI was found to be in a mainly truncated and inactive form in lavage fluid of patients with ARDS [7], and TFPI activity was reduced in patients with pneumonia, suggesting rh-TFPI may be of therapeutic value in this setting [12, 42]. The ensuing CAPTIVATE trial, specifically designed to investigate the effect of rh-TFPI in severe CAP, demonstrated attenuation of coagulation, however failed to show a beneficial effect on outcome [23]. In these clinical trials anticoagulant agents were administered systemically, consequently increasing the incidence of bleeding complications. Indeed, the OPTIMIST trial reported more adverse events with bleeding in patients treated with rh-TFPI than in placebo treated patients (24% versus 19%) [14], which may have counterweighed potentially favorable effects. Recently, the first small studies of nebulized heparin treatment have been conducted in patients with ARDS and report inconsistent anticoagulant effects [18, 43, 44].

There are several limitations to our experimental study. The chosen dosage for nebulization was based on data from previous studies combined with a calculation of the efficacy of the nose-only exposure system [12, 13]. Importantly, rh-TFPI was only detectable in the pulmonary compartment and did not influence systemic TFPI activity. However, lower concentrations of rh-TFPI may suffice and yield the same anticoagulant effect, whereas higher treatment concentrations may be needed for local anti-inflammatory or antibacterial effects. Furthermore, we did not investigate functional endpoints, such as alveolar gas exchange, nor the effect on overall outcome. In our study we investigated the effect of pre-treatment with local rh-TFPI in the absence of antibiotic treatment, which does not resemble the clinical situation and may elicit different effects. For instance, in a murine model of ongoing pneumonia, delayed rh-TFPI treatment inhibited accumulation of neutrophils in lung tissue and reduced cytokine and chemokine levels, suggesting that TF-mediated coagulation might only influence inflammation during an ongoing procoagulant/proinflammatory response, and this effect became only apparent without concurrent antibiotic treatment [13]. As such, a pilot phase II trial investigating post-pneumonia treatment with concurrent antibiotic treatment would be necessary to translate the results to the clinical practice. In addition, it would be of interest to combine nebulization of rh-TFPI with other anti-inflammatory and anticoagulant agents that are frequently used in a clinical setting, such as heparin or corticosteroids. Of note, also during non-infectious lung injury, for instance induced by mechanical ventilation, pulmonary coagulation is enhanced [45], which implies patients may benefit from nebulization with rh-TFPI in this setting. Notably, delayed rh-TFPI treatment showed a modest antibacterial effect in the lung, and a growth inhibiting effect on *S*. *pneumoniae* in the presence of human serum in vitro was observed [13]. Recent studies describe antimicrobial activity of C-terminal peptides of the rh-TFPI molecule against several pathogens via the complement system [46, 47]. In addition, rh-TFPI in combination with antibiotic treatment improved survival in mice challenged with Gram-negative bacteria [48]. In our study pre-treatment with nebulized rh-TFPI did not influence bacterial loads in the lung or plasma in our model. Therefore, it will be of interest to investigate the therapeutic value of delayed treatment with nebulized rh-TFPI or C-terminal derived peptides, and the effect of concomitant antibiotic treatment, mimicking the clinical situation.

## Conclusions

In conclusion, the TF-mediated procoagulant environment within the lung compartment during pneumonia provides a rationale for local treatment with rh-TFPI. We show that local treatment with rh-TFPI has clear anticoagulant effects, which are restricted to the lung compartment, thereby minimizing the risk of potential harmful adverse effects. Finally, local treatment with rh-TFPI did not induce local bleedings.

## Supporting Information

S1 Fig Nebulization with rh-TFPI does not influence lung weight or cytokine/ chemokine levels in lungs in rat pneumococcal pneumonia. (TIF)
