# Supplementary figures and images for: Feasibility and Safety of Local Treatment with Recombinant Human Tissue Factor Pathway Inhibitor in a Rat Model of Streptococcus pneumoniae Pneumonia
Source: PLoS One. 2015 May 18;10(5):e0127261. doi: 10.1371/journal.pone.0127261 (PMC4436292; doi:10.1371/journal.pone.0127261)

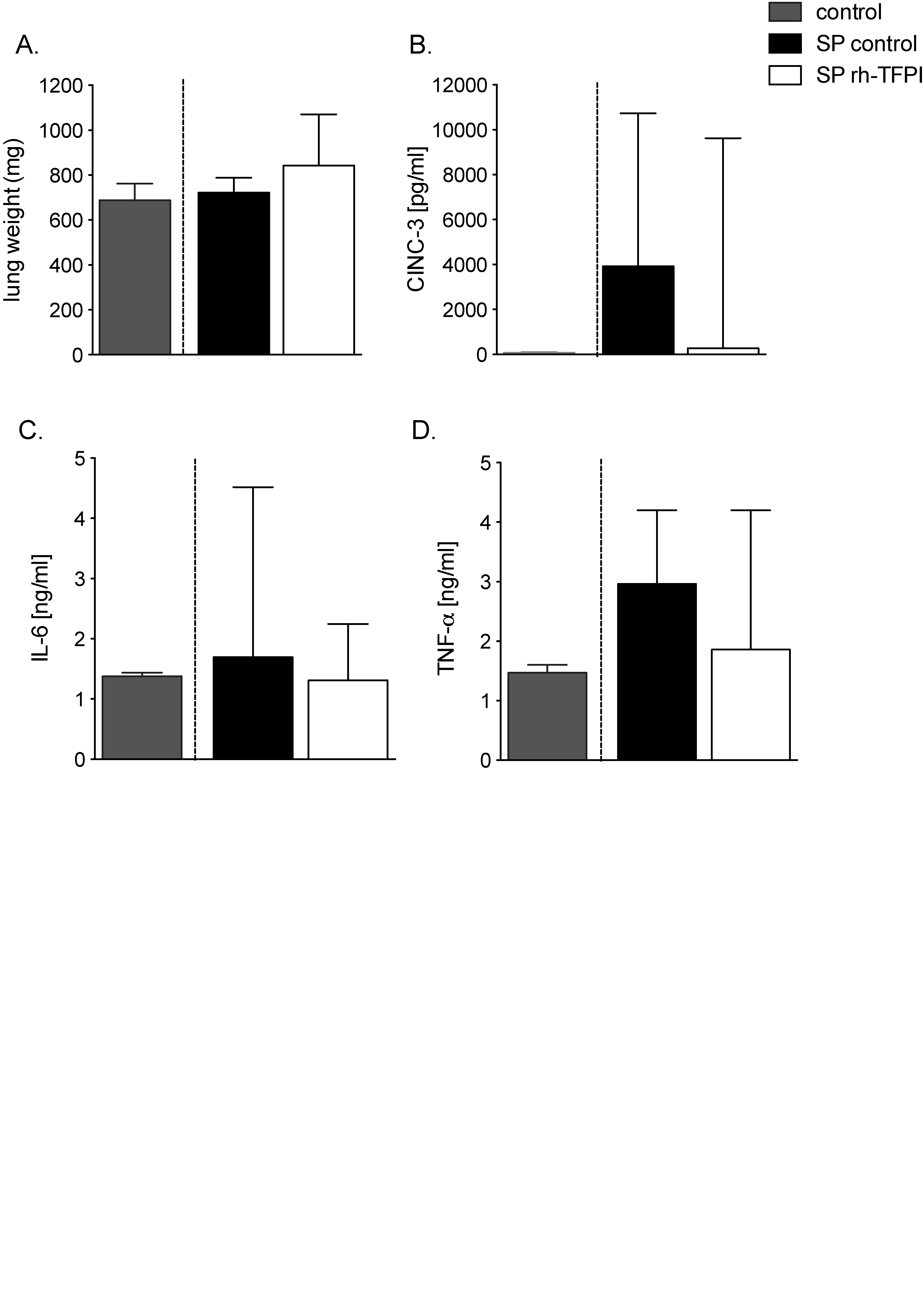

Supplement: S1 Fig — (TIF) [file pone.0127261.s001.tif]
